# Can excreted thiocyanate be used to detect cyanide exposure in live reef fish?

**DOI:** 10.1371/journal.pone.0196841

**Published:** 2018-05-30

**Authors:** Nancy E. Breen, Julie Lowenstein, Rebecca Metivier, Lawrence Andrade, Andrew L. Rhyne

**Affiliations:** 1 Department of Chemistry and Physics, Roger Williams University, Bristol, RI, United States of America; 2 Department of Biology, Marine Biology and Environmental Science, Roger Williams University, Bristol, RI, United States of America; 3 Dominion Diagnostics, North Kingstown, RI, United States of America; Duke University Marine Laboratory, UNITED STATES

## Abstract

Cyanide fishing, where a solution of sodium or potassium cyanide is used to stun reef fish for easy capture for the marine aquarium and live fish food trades, continues to be pervasive despite being illegal in many countries and destructive to coral reef ecosystems. Currently, there is no easy, reliable and universally accepted method to detect if a fish has been exposed to cyanide during the capture process. A promising non-invasive technique for detecting thiocyanate ions, the metabolic byproduct excreted by exposed fish, has been reported in the literature. In an effort to validate this method, four cyanide exposure studies on *Amphiprion ocellaris* (common clownfish) were carried out over three years. Fish were either exposed to the same (25 ppm) or twice the concentration (50 ppm) as the previsouly published method. Over 100 water samples of fish exposed to cyanide were analyzed by reverse phase HPLC with a C_30_ column treated with polyethylene glycol and UV detector operating at 220 nm. No thiocyanate was detected beyond the analytical standards and positive controls prepared in seawater. As an alternate means of detecting thiocyanate, water samples and thiocyanate standards from these exposures were derivatized with monobromobimane (MBB) for LC-MS/MS analysis. Thiocyanate was detected in standards with concentrations as low as 0.6 μg/L and quantified to 1 μg/L, but thiocyanate could not be detected in any of the water samples from fish exposed to cyanide with this method either, confirming the HPLC results. Further, we calculated both the mass balance of thiocyanate and the resultant plausible dosage of cyanide from the data reported in the previously published method. These calculations, along with the known lethal dosage of cyanide, further suggests that the detection of thiocyanate in aquarium water is not a viable method for assessing fish exposure to cyanide.

## Introduction

The marine aquarium trade (MAT) and the live food fish trade (LFFT) continue to flourish in the Indo Pacific region, despite the use of fishing methods destructive to the coral reef ecosystem where these fish are captured. Cyanide has been used by the MAT and LFFT as a paralytic agent to stun and capture fish in the Philippines and Indonesia for over 50 years [1,2]. In the 1970s, as the demand for live food fish in Hong Kong and China grew, the use of cyanide spread throughout the Indo Pacific [3]. Cyanide continues to be used despite being illegal and it has been estimated that over one million kilograms of cyanide have been used in the Philippines since the 1960s [4]. Due in part to this destructive practice, as well as other factors, over 95% of the reefs of this region have been labeled as at risk, with nearly half in the high or very high threat category [5]. To combat this threat, efforts have been focused on developing the ability for governments and trade organizations to detect if fish have been captured using cyanide [6].

Cyanide fishing is used to capture reef fish because it is an easy and inexpensive method to capture high value reef fish alive [2]. It also allows fishermen to catch fish that hide in crevices and are more difficult to catch. Fishermen place sodium cyanide or potassium cyanide tablets in seawater filled squirt bottles that divers take to the reef. The solution is squirted at the target species, stunning the fish, and making collection easy [3]. The practice is non-selective and both the targeted fish and non-targeted species, as well as the live coral reef, are exposed to the poison. Many exposed fish receive an acute dose and die in the capture or transport process. Cyanide exposure is known to cause coral bleaching in reefs, as cyanide disrupts the symbiotic relationship between the coral polyp and the zooxanthelle which provide the coral with nutrients [7]. Additionally, coral colonies are often broken off in order to capture the hiding fish, ruining the three dimensional structure of the reef habitat. Despite being illegal, cyanide fishing is still commonly practiced by fishermen in poor, rural communities who rely on the trade for their livelihood [2,8].

Some attempts to curtail the practice have been based on the detection of cyanide or its metabolites in captured fish. Detection methods vary by the ease of use, the need to sacrifice the fish, the detection limit, and the robustness of the method. These methods include colorimetry, titrimetry, ion-selective electrodes, bio-sensors, gas chromatography and high performance liquid chromatography (HPLC) [9,10]. The most commonly used method is the cyanide detection test (CDT) developed by the International Marinelife Alliance (IMA) at the request of the Philippine Department of Agriculture Bureau of Fisheries and Aquatic Resources (BFAR). In the 1990s eight CDT laboratories were established throughout the Philippines to use the newly developed test. More than 48,000 fish were tested over 8 years and 25% of them tested positive for cyanide [6]. It is believed that the test served as a deterrent as the number of fish testing positive for cyanide declined during the testing period [11,12]. Today there are fewer CDT laboratories currently active in the Philippines and the test itself has come under scrutiny because of its susceptibility to false positives. The test also requires using larger fish or pooling small fish (minimum 10 grams), and sacrificing the fish. The analysis itself is very laborious taking 2–3 hours per sample [11].

In 2012, a non-invasive method for identifying fish exposed to cyanide appeared to be a promising alternative to the CDT test [13]. The authors reported that the excreted metabolite thiocyanate could be detected in holding water of fish exposed to cyanide based on the analytical method developed by [14]. Briefly, utilizing reverse phase HPLC with a C_30_ column treated with polyethylene glycol and an optical fiber detector, the authors reported detection of thiocyanate in seawater in which the fish had been held. The theory behind the detection test proposed by [13] was that after exposure to cyanide, the major metabolic pathway is the conversion of cyanide to thiocyanate by the enzyme thiosulfate sulfurtransferase. The conversion rate in humans is reported to be around 80% [15,16]. While the half-life of cyanide for acute exposure in mammalian species is short, varying from 0.34 to 1.28 hours, that for thiocyanate is much longer, varying from 4.95 to 192 hours [16]. In the freshwater fish rainbow trout (*Oncorhynchus mykiss*), the blood plasma level of thiocyanate reaches a maximum concentration of 78.9 mg/L after 16 days upon continual exposure to 39.8 mg/L of thiocyanate. When allowed to depurate, the half-life for clearance was reported to be 2.02 or 2.36 days depending on the statistical model used to fit the data [17].

Vaz et al. [13] pulse exposed captive bred clownfish (*Amphiprion clarkii*) for 60 seconds to 12.5 or 25 mg/L cyanide and held the fish in 1.5 L of synthetic seawater for 28 days post exposure to depurate. Water samples were collected daily and the water from each jar was replaced daily. They reported that excreted thiocyanate concentrations peaked at 6.96 +/- 0.03 μg/L or 9.84 +/- 0.03 μg/L for low (12.5 mg/L) and high (25 mg/L) exposures respectively. Notably, they reported that thiocyanate was detected at near peak values for the entire 28-day period with no evidence of a decrease in the concentration of the excreted thiocyanate reported (S1 File.). The authors suggested that marine fish unlike freshwater fish must retain the thiocyanate in the bloodstream longer due to physiological differences. Further, they reported extremely low variation within treatments (i.e. 0.3–0.4%), suggesting the test is highly repeatable.

In a similar experiment, *Amphiprion frenatus* (tomato clownfish) and *Pterapogon kauderni* (Banggai cardinalfish) were exposed to 12.5 mg/L cyanide for 60 seconds or 50 mg/L cyanide three times for 5 seconds, and allowed to depurate for 14 days in 1.225 L of natural filtered seawater [18]. Water was changed daily, and sampled on days 2, 5, 8, 11 and 14. Thiocyanate was detected in positive controls (spiked artificial seawater samples) using the HPLC method developed by [13]. No thiocyanate peak was observed in holding water from any fish exposed to cyanide [18]. Additionally, the authors reported an experiment carried out in Banyuwangi, East Java, Indonesia, which is a known cyanide fishing hot spot, using a protocol similar to [13]. Live reef fish (n = 37) comprising 4 different species were procured from local fishermen. The fish were held in jars for 6 days and water samples were collected daily from the jars containing these fish and were tested for thiocyanate. Again, no thiocyanate was detected in any of the water collected from the fish, but thiocyanate was detected in the positive controls [18].

While no experimental confirmation has yet been reported to independently validate [13], these same authors reported findings from a survey of imported fish, suggesting that 14% of marine aquarium fish imported into the EU were displaying physiological signs of cyanide exposure [19].

The primary goal of our study was to validate the original findings of [13] and extend their work to a different, but similar species, *Amphirion ocellaris*. The work reported here should be viewed as a complementary study and not an exact duplication of [13]. Four exposure experiments were carried out over a three-year period, varying saltwater sources (natural and synthetic) and cyanide sources (sodium and potassium salts). Exposure times and cyanide concentrations varied only minimally from the conditions described by [13]. We did not use optical fiber detection, but our HPLC-UV method was adapted from the method reported by [19,20] and was capable of detecting thiocyanate in analytical standards to 4 μg/L, which is consistent with the previous published methods. Further, we report on an improved method for the detection of thiocyanate in seawater adapting the LC-MS/MS derivatization method developed by [21] to corroborate our HPLC-UV findings.

## Materials and methods

### Test species and cyanide sources

*Amphiprion ocellaris* have been extensively cultured at Roger Williams University, Bristol, Rhode Island, USA for over 10 years and laboratory reared specimens were used for all exposure experiments ensuring no previous cyanide exposure. The initial goal of the study was to conduct a simple control / treatment study to validate [13]. Due to challenges replicating [13] we expanded our scope and three additional separate exposure experiments were carried out with treatment conditions being summarized in Table 1. Only the first exposure was a traditional experimental design, all other exposures were designed to minimize animal use and to evaluate the validity of the method. In all tests, we followed the general exposure protocol employed by [13]. The cyanide used was from a 2.5% stock solution of sodium cyanide (LabChem) or a stable isotopic ^13^C labeled potassium cyanide salt (Sigma) and was diluted or dissolved appropriately to yield the desired concentration. After the pulsed exposure to cyanide (25 or 50 mg/L) for 60 seconds, the fish were dipped successively into two seawater rinse baths. After rinsing, fish were allowed to recover in a 20L aerated vessel until they regained balance (18–26 minutes) then placed in glass jars filled with 1.0 L seawater with a salinity of 35 (either natural or artificial depending on exposure). Each jar was supplied air via an airstone and placed in a climate controlled room set to 26°C with a photoperiod of 12 hours of light and 12 hours of dark. The fish were allowed to depurate for 14–30 days depending on the trial. During the depuration period, 10 mL water samples were collected, then fish were fed to satiation with flake food and the water was replaced daily. Water samples were stored at -80°C or -20°C until analyzed.

**Table 1:** Summary of cyanide exposure experiments at Roger Williams University. Numbers indicate the experimental units measured during each exposure. Control 1 indicates a negative control, a fish with no exposure. Control 2 is a positive control, and has a fish present with thiocyanate spiked to 20 μg/L daily with each water change. Control 3 has thiocyanate at 20 μg/L aerated with no fish present. Exposures 3 and 4 used two different brands of widely available synthetic seawater.

Exposure	Date	Water	Method	Duration	25 mg/L for 60 s	50 mg/L for 60 s	Control 1	Control 2	Control 3
1	October 2014	Filtered seawater	A	28 days	10	-	10	-	-
2	January 2015	Filtered seawater	A	28 days	5	-	-	-	-
3	June 2016	Instant Ocean^®^	A,B	30 days	-	7	1	1	1
4	January 2017	Tropic Marin^®^	A,B	14 days	-	4	2	2	1

Method A, HPLC-UV; Method B, LC-MS/MS; Exposure 4 used labeled ^13^C potassium cyanide

### Thiocyanate analysis

Thiocyanate standards and water samples from all exposures were analyzed by HPLC-UV [20] using a Waters 515 pump, a Jasco UV-1575 single wavelength detector operating at 220 nm and a Peak Simple data acquisition system (SRI Instruments). The column (Devosil 5 μm C_30_-UG, 150 x 4.6 mm id) was modified with 5% polyethylene glycol (PEG). The column was rinsed with the PEG solution for at least 30 minutes with a flow rate of 0.3 mL/min and then with the mobile phase for 1 hour or until the pressure stabilized prior to use for analysis. The mobile phase consisted of 300 mM sodium sulfate and 50 mM sodium chloride for the first two exposure studies or 300 mM sodium sulfate for the second two exposure studies. Injections were carried out manually using a Rheodyne 7125 injector with a 20 μL sample loop. Analytical standard solutions of thiocyanate^-^ (2, 4, 6, 8 10 and 20 μg/L in seawater) were used to generate a calibration curve. The retention time of thiocyanate was stable on a day to day basis, but did vary between 4.3 and 5.8 minutes over the 4 year period if different Devosil columns were used. Our detection capabilities did not differ by column.

Thiocyanate was also analyzed using LC-MS/MS following the method of [20]. Samples were first derivatized with monobromobimane to form a bimane-thiocyanate adduct in seawater. The LC-MS/MS system used was comprised of a Waters Acquity IClass UPLC and a AB Sciex API5500 QTrap mass spectrometer operating in negative ion mode. Samples were injected onto a Phenomenex Kinetex XB-C18 1.7 μm 2.1 x 50 mm column (PN 00A-4498-AN) held at 40°C. The analytes and internal standard were eluted using a gradient chromatographic method with 10mM ammonium formate as mobile phase A, and 10mM ammonium formate in methanol as mobile phase B. The flow rate was 0.25 mL/minute with a total run time of 6 minutes.

Analyte validation and quantification was achieved by calculation of peak area ratio (analyte/internal standard) and subsequent concentration from a linear weighted (1/x) regression of calibration standards. Table 2 presents the selected reaction monitoring (SRM) precursor → product ion transitions used. To accept a calibration standard, the calculated concentration must deviate ≤ 10% from the nominal concentration. The analyte response at the lower limits of quantification and detection (LLOQ and LOD) must have a signal-to-noise ratio (S/N) ≥ 10 and 3 respectively, as calculated by the LC-MS/MS data acquisition and processing software, MultiQuant™ 3.0. The major modification to the procedure developed by [20] was to lower the concentration of internal standard from 100 μM (8300 μg/L) of NaS^13^C^15^N to 220 μg/L of S^13^C^15^N. Standard solutions of thiocyanate prepared in seawater (0.1–50 μg/L) were used to generate a calibration curve. Samples were prepared at Roger Williams University and then sent out for analysis at Dominion Diagnostics Laboratory in North Kingston, RI.

**Table 2:** Transitions used for selected reaction monitoring (SRM) for LC-MS/MS data acquisition and processing used in exposures 3 and 4.

Thiocyanate Isotope	Parent Ion	Product Ion	Transition ID
SCN	248.0	111.0	SCN Primary/Quant
SCN	248.0	124.1	SCN Secondary/Qual
S^13^CN	249.0	111.0	SCN Primary/Quant
S^13^CN	249.0	125.1	SCN Secondary/Qual
S^13^C^15^N	250.0	111.0	SCN IS Primary/Quant
S^13^C^15^N	250.0	126.1	SCN IS Secondary/Qual

## Results and discussion

**Fig 1:**
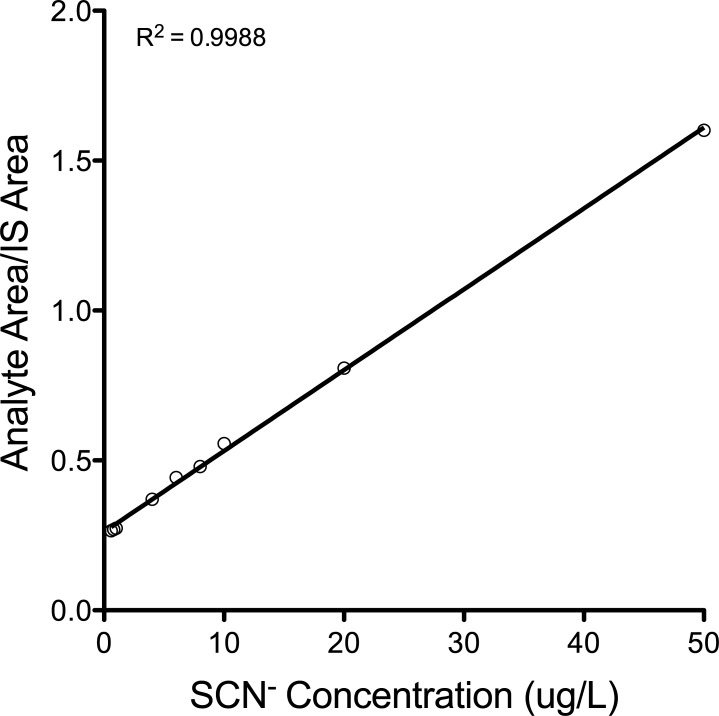
Typical standard curve of LC-MS/MS measurement of S13CN in seawater.

Four cyanide exposure experiments on *Amphiprion ocellaris* (common clownfish) were undertaken over a three-year period (2014–2017). In all exposures, regardless of seawater or cyanide source, the fish behaved similarly to what was reported in other studies [22,23]. Following exposure, fish were always paralyzed and took between 18–26 minutes to recover balance and the time to recovery appeared to be related to cyanide concentration and exposure time. The fish did not feed for 3–4 days after exposure. Over 100 samples collected from aquaria water containing fish exposed to cyanide at 25 mg/L or 50 mg/L were analyzed from these exposure experiments. All attempts to detect thiocyanate excreted by fish exposed to cyanide proved to be unsuccessful using both the HPLC/UV method and the LC-MS/MS method. The former method is capable of detecting 4 μg/L in seawater standards and the latter has an LOD of 0.6 μg/L in seawater standards. A typical calibration curve for the LC-MS/MS method is shown in Fig 1.

**Fig 2:**
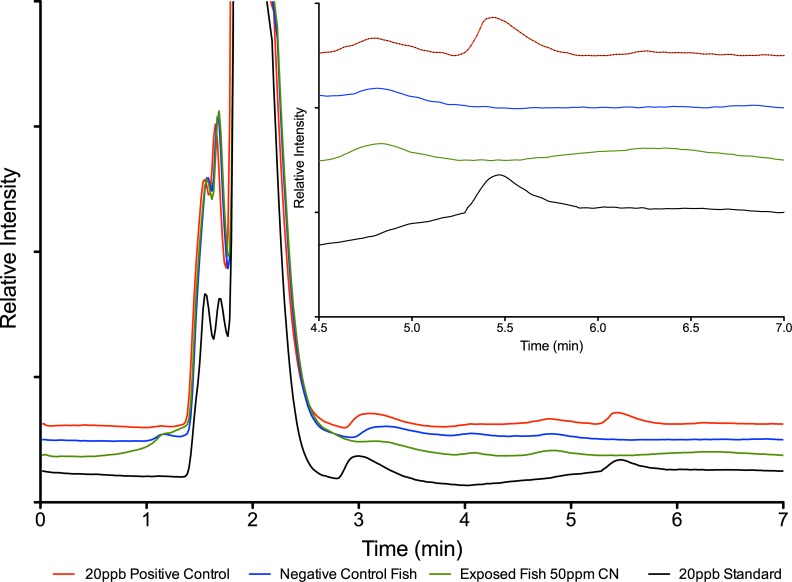
Typical chromatograms of HPLC-UV method. From bottom to top, a 20 μg/L standard, a water sample from a control fish (no cyanide exposure), a water sample from an exposed fish from 01-13-2017 and a water sample positive control 3, spiked to 20 μg/L thiocyanate produced by the HPLC method. The 20 μg/L standard and spiked water sample show a clear thiocyanate peak with a retention time of about 5.5 minutes (see dashed box). The control fish and exposed fish show no peak at the same retention time. Graphs are stacked to show detail.

Thiocyanate was routinely detected in the positive controls where the jars were spiked to contain 20 μg/L thiocyanate and aerated for 24 hours. In the positive control (Control 3) that consisted of aerated seawater and 20 μg/L thiocyanate only (no fish present) recovered concentrations were 19.8 +/- 2.0 μg/L (n = 5) after 24 hours. In the positive control (Control 2) that consisted of aerated seawater with 20 μg/L thiocyanate and a fish not exposed to cyanide, the thiocyanate concentration after 24 hours was found to be 14.6 +/- 1.3 (n = 5). The statistically significant (p = 0.00021) lower level of thiocyanate in the positive Control 2 indicates the fish absorbs some of the thiocyanate present in the water. While we were able to apply the HPLC method successfully to detect thiocyanate to 4 μg/L in our controls, it proved to be cumbersome and lacked precision. The coefficient of variation was 6.7% for 5 repeated injections of 20 μg/L thiocyanate standard. Retention times varied slightly day-to-day and standards had to be run at least daily to confirm the thiocyanate retention time for both qualitative and quantitative evaluation. Despite these issues, the method can be used to test for the presence of trace amounts of thiocyanate. Typical chromatograms are shown in Fig 2 in water samples of controls, standards, and treatment fish analyzed by HPLC with UV detection.

Because we were unable to reproduce the results of [13] over the course of the initial exposure experiment, a number of the experimental parameters were varied in an effort to elucidate the cause of our inability to detect thiocyanate in exposed fish. Two cyanide sources were used, at two different doses (25 mg/L and 50 mg/L). A major difference between our initial trial and that of [13] was the water used, they used synthetic seawater and we used filtered natural seawater. To account for this difference, we did not use filtered seawater for exposures 3 and 4 but rather, Instant Ocean (exposure 3) and Tropic Marin (exposure 4) prepared water. Both salts are commercially available synthetic seawater salts and the latter was used by [13]. The artificial seawater was made from reverse osmosis plus deionized water (18 ohms). We also tested analytical grade seawater formulated from laboratory salts and made with HPLC grade water (Sigma) for several water changes in exposure 4. Similar to [18], excess iron (III) nitrate was added to thiocyanate standards to see if the presence of multivalent cations could interfere with thiocyanate detection, but the thiocyanate peak remained unchanged in the chromatogram. In exposure 4, the pH was carefully monitored with only small variations observed, but it was adjusted to 8.2 prior to analysis with strong base (6M NaOH) for several samples. None of these variables proved to be the key to detecting thiocyanate in water samples collected from fish exposed to cyanide.

**Fig 3:**
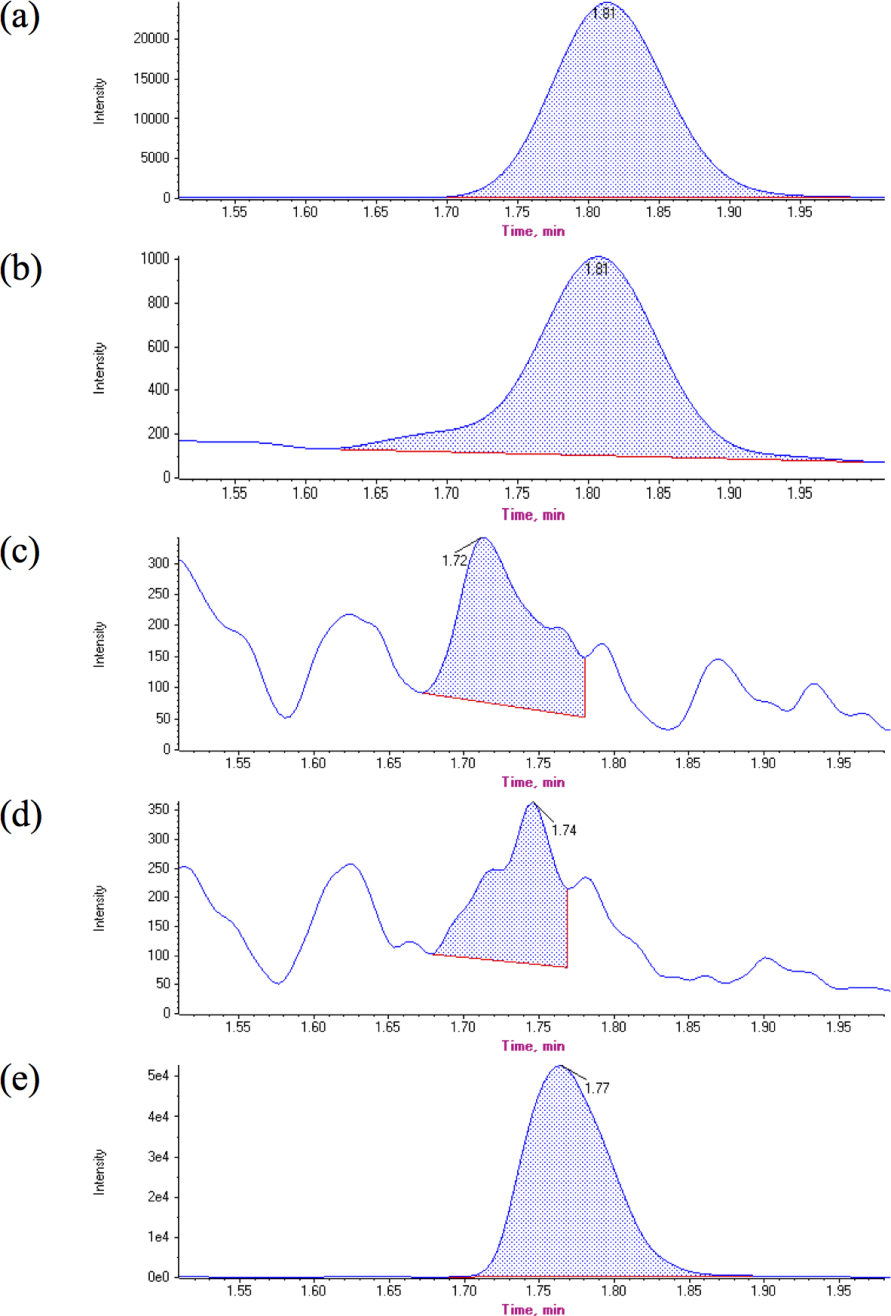
Typical chromatograms for LC-MS/MS analysis. The chromatograms represent the signal response for the MRM transition 248.0 -> 111.0 for the thiocyanate-bimane derivatization product. 20 microgram per liter (b) 1 microgram per liter (c) Control 1 (d) exposed fish and (e) control 3.

When the fourth attempt to detect thiocyanate using the HPLC/UV method proved unsuccessful, the water samples collected from exposure 3 and 4 were re-analyzed using LC-MS/MS, a more specific and sensitive methodology. The LC-MS/MS limits of quantification and detection (LLOQ and LOD) were 1.0 and 0.6 μg/L, respectively. Over 60 samples were analyzed using this method but no thiocyanate was detected in water collected from fish exposed to cyanide for samples from day 2 to day 12 post exposure. Typical chromatograms for these analyses are shown in Fig 3. From a chromatographic standpoint, this method was much more sensitive, robust, and efficient. Run times were shorter, and because of the derivatization procedure there was no variation in retention time at all and no column pre-treatment was required. However, the instrumentation and isotopically labeled reagents are more expensive when compared to HPLC-UV analysis.

It is clear that HPLC with UV detection is capable of detecting thiocyanate present in seawater at concentrations at or above 4 μg/L and that the LC-MS/MS method is capable of detecting thiocyanate present in seawater at concentrations at or above 0.6 μg/L. According to [13], thiocyanate levels were observed to be on average 5.70 μg/L over the 28 days reported for fish in the 60 second exposure at 12.5 mg/L cyanide. They also reported an average fish weight of around 2 g and their tanks contained 1.5 L of water. From these reported values, each fish secreted on average $\left(1.5\;L)\left(\frac{5.70\mu g}{L}\right)(28\;days\right)$ = 239 μg of thiocyanate during the 28-day interval. It is worth noting that while an increase of excreted thiocyanate was observed over the first few days post exposure in [13], no decrease in thiocyanate excretion level was observed, even after 28 days, presumably indicating there was still a reservoir of thiocyanate within the fish. Therefore, the total amount of thiocyanate contained in the fish would be greater than 239 μg. If a fish contained 239 μg of thiocyanate, it would have to absorb a minimum of 107 μg of cyanide, assuming all cyanide absorbed by the fish was excreted as thiocyanate. The conversion to thiocyanate is the major cyanide detoxification pathway in vertebrate animals, responsible for about 80% of the cyanide absorbed, provided there are sulfur donors. It is likely that the amount of cyanide absorbed by the fish would have been larger (134 μg), however, since the actual conversion ratio is not known for marine fish, we will not adjust our value upwards but rather keep the 107 μg as a more conservative value. Therefore, in order for the average reported value of 5.70 μg/L of thiocyanate recovered from the depurated fish to be valid, the cyanide dose received by the fish (average mass 2 g) would have to be at least 53.6 mg/kg in the 12.5 mg/L exposure. Similarly, the cyanide dose would be 78.5 mg/kg for the for the 25 mg/L exposure.

**Fig 4:**
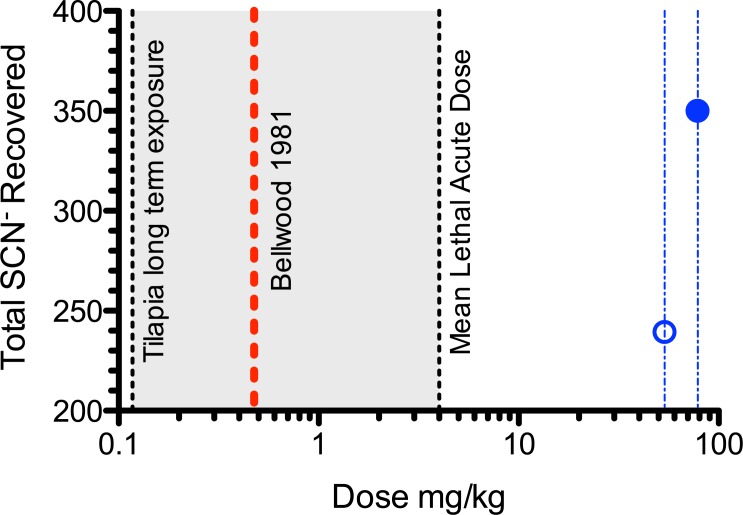
The minimum dose of cyanide required to achieve reported thiocyanate recovered over a 28 day depuration period reported by [13]. Blue circles are total thiocyanate (open circle is low dose and closed circle is high dose) reported and corresponding dose (blue dotted lines) reported by [13]. Red dotted line is the calculated dose from a radio labeled exposure study [20], see supplemental materials for calculations. Dashed lines are literature values depicting LC50 after 96 hour exposure for Tilapia [20] and mean lethal acute dose (4 mg/kg) an animal could receive and survive [15]. Gray shaded area indicates a plausible dose.

It seems improbable that these doses could be achieved from the concentration of cyanide in the bath that the fish were exposed to. The rate of uptake and dose after acute exposure of cyanide in marine fish is poorly understood [1,11,22]. The best estimate for the concentration of cyanide taken up by marine fish during an exposure is a small preliminary study conducted on the damselfish *Neopomacentrus violascens* [22]. Three damselfish were exposed to 1.1 mg/L of radio labeled ^14^C cyanide until anaesthetized plus 30 seconds. The brain, gills, liver, intestine, spleen, and stomach were removed and the radioactivity was measured in each organ. From the data reported, the average total amount of cyanide accumulated was 1.04 μg, giving an average dose of 11.3 mg/kg in these organs where cyanide is known to accumulate. The mass of the fish exposed was estimated to be between 1.6 to 2.2g (per. comm. David Bellwood), which yields a dose between 0.65 and 0.47 mg/kg of fish, respectively (S1 File.). In a different study, freshwater Nile tilapia were exposed to 0.129 mg/L cyanide for 24 hours, a much lower dose and a much longer interval than [13]. In this study, the cyanide was found to be of 0.115, 0.094, 0.107 and 0.118 mg/kg in the gills, liver, muscles and blood at 1-hour post exposure and the observed cyanide concentrations steadily decreased with time post exposure [24]. These values are in reasonable agreement with each other given the differences in species and experimental protocol and represent the best estimate of cyanide uptake in fish. In both cases, the observed cyanide dose is much less than the estimated dose needed (53.6 and 78.5 mg/kg) to excrete the amount of thiocyanate reported by [13] and significantly lower than the typical LD50 as discussed below (Fig 4). More simplistically, if cyanide was allowed to freely distribute throughout the volume of the fish and metabolism is not considered, the maximum amount of cyanide that an average sized fish (i.e. the average volume of a 2 g fish is 2 mL) could amass in a 12.5 ppm cyanide solution is 25 μg. This value is much less than the amount of cyanide that would need to be absorbed by a 2 gram fish to excrete 5.70 μg/L of thiocyanate into a 1.5 L tank for 28 days. Unless cyanide is supersaturated within saltwater fish under the conditions described in [13], it does not seem possible for a fish to absorb the amount of cyanide needed to excrete the amount of thiocyanate reported by [13]. Moreover, when considering the toxicity and metabolism of cyanide, this dose seems even less feasible.

Of course, even if the fish could absorb cyanide at these levels, could they survive? For mammals, fish and some invertebrates, the toxic effects of cyanide occur by interference with aerobic metabolism, when cyanide blocks terminal electron transfer by binding to cytochrome oxidase [21]. The doses required by [13] (53.6 and 78.5 mg/kg) appear to be well above the effective acute lethal dosage of cyanide in most animal species of 4mg/kg [15]. The MSDS for sodium cyanide reports an acute oral exposure LD50 to be 6.44 mg/kg NaCN for a rat and an acute dermal exposure LD50 of 10.4 mg/kg NaCN for a rabbit. These doses correspond to 3.42 mg/kg and 5.52 mg/kg in cyanide. For fish, cyanide is absorbed through the gills or the intestine, but one can expect lethal doses to be similar in all species as the mechanism of action is similar. The LC50 for Nile tilapia after 96 hours exposure was reported to be 0.387 mg/L [24]. These dosage data, which are summarized in Fig 4, suggest that even if the exposed fish could absorb enough cyanide to excrete thiocyanate at the detectable levels reported by [13], they would not survive the exposure.

## Conclusions

The detection of cyanide exposure from cyanide fishing is an important step in combating illegal and destructive fishing activities associated with the LRFF and MAT. For over 30 years, efforts have been made to develop testing methods to detect cyanide exposure in reef fish. A simple test like that proposed by [13] to detect if a fish was exposed to cyanide prior to capture could be pivotal in policing the MAT industry. In this study, we found that after multiple exposure experiments with negative results that if excreted thiocyanate is present in aquaria water from *Amphiprion ocellaris* exposed to cyanide, it must be at concentration below 0.6 μg/L. Furthermore, our mass balance calculation of [13] suggests that for a 2 gram fish to be able to excrete an average thiocyanate concentration of 5.70 or 8.34 μg/L over 28 days would require much too high a dose of cyanide for the fish to survive, greatly exceeding the lethal dose in all known animals. While our results are in agreement with [18], we could not independently validate the method proposed by [13] using *Amphiprion ocellaris* as a test species. While detecting cyanide exposure via excreted thiocyanate in aquaria water seemed promising, we have yet to find conditions and/or fish species that can validate the results obtained by [13]. The usefulness of this method as a tool to combat cyanide fishing seems questionable.

There is an urgent need for a rapid, simple, and cost effective test to detect fish collected with cyanide beyond that developed by IMA [11]. This need can only be met by first understanding the physiology and biochemistry of marine fish exposed to cyanide in a controlled laboratory environment. Some 35 years after David Bellwood first conducted his pilot study [22], we have yet to answer the questions, what is the typical dose of cyanide received by a marine fish, what is the LD50 for various marine fish species, and most importantly, how long does cyanide or the metabolites of cyanide exposure remain in a given fish. Our understanding of the effects of cyanide exposure in fish has not advanced much beyond that reported in the second half of the 20^th^ century. Unfortunately, we have not been able detect thiocyanate in aquaria water of *Amphiprion ocellaris* exposed to cyanide using HPLC-UV or LC-MS/MS. We hope in reporting our findings that it will provide the stimulus needed for the MAT industry to look for alternate methods to detect cyanide exposure in captured fish and that can lead to the development of certification schemes for cyanide free supply chains.

## Supporting information

S1 File Calculations of cyanide dosage from the literature. Bellwood (1981) radioactive cyanide exposure and Vaz et al. (2012) accumulated thiocyanate calculations.
(XLSX)
