# Correction: Can excreted thiocyanate be used to detect cyanide exposure in live reef fish?

**DOI:** 10.1371/journal.pone.0205552

**Published:** 2018-10-04

**Authors:** Nancy E. Breen, Julie Loewenstein, Rebecca Metivier, Lawrence Andrade, Andrew L. Rhyne

The second author's name is spelled incorrectly. The correct name is: Julie Loewenstein. The correct citation is: Breen NE, Loewenstein J, Metivier R, Andrade L, Rhyne AL (2018) Can excreted thiocyanate be used to detect cyanide exposure in live reef fish? PLoS ONE 13(5): e0196841. https://doi.org/10.1371/journal.pone.0196841
